# Pea Starch and Pea Fiber—A Review of Functional Properties, Sensory Characteristics, and Value‐Added Uses

**DOI:** 10.1111/1541-4337.70419

**Published:** 2026-02-14

**Authors:** Mathias Johansson, Klara Nilsson, Madeleine Jönsson, Karin Wendin, Anna Ström, Maud Langton

**Affiliations:** ^1^ Department of Chemistry and Chemical Engineering Chalmers University of Technology Gothenburg Sweden; ^2^ Department of Molecular Sciences Swedish University of Agricultural Sciences Uppsala Sweden; ^3^ Department of Food and Meal Science Kristianstad University Kristianstad Sweden; ^4^ Department of Food Science University of Copenhagen Frederiksberg Denmark

## Abstract

The utilization of plant‐based ingredients, such as legumes, is gaining popularity because of consumer demand for sustainably sourced, high‐protein foods, and an increased number of consumers reluctant to consume animal‐based products. Legumes are often cultivated for their protein but are also rich in starch and fiber. Furthermore, during protein fractionation, large side streams composed mainly of starch and fiber are produced. To promote efficient use of biomass, it is essential to find effective ways of repurposing these side streams. Understanding the functionality of the side streams allows the identification of alternative uses that offer economic and environmental benefits. One crop that is gaining attention and technical use as a sustainable, plant‐based protein source is pea (*Pisum sativum* L.). This review provides an overview and establishes new connections between the physicochemical properties of pea starch and pea fiber, available treatment methods for further functionalization, and the influence of pea starch and pea fiber on different foods and their sensory properties and product performance across various applications. Challenges and knowledge gaps are also identified, for example, the correlations between chemical and sensory properties and how changes in starch and fiber functionality affect the textural and sensory properties of the final food product.

## 1 Introduction

**FIGURE 1:**
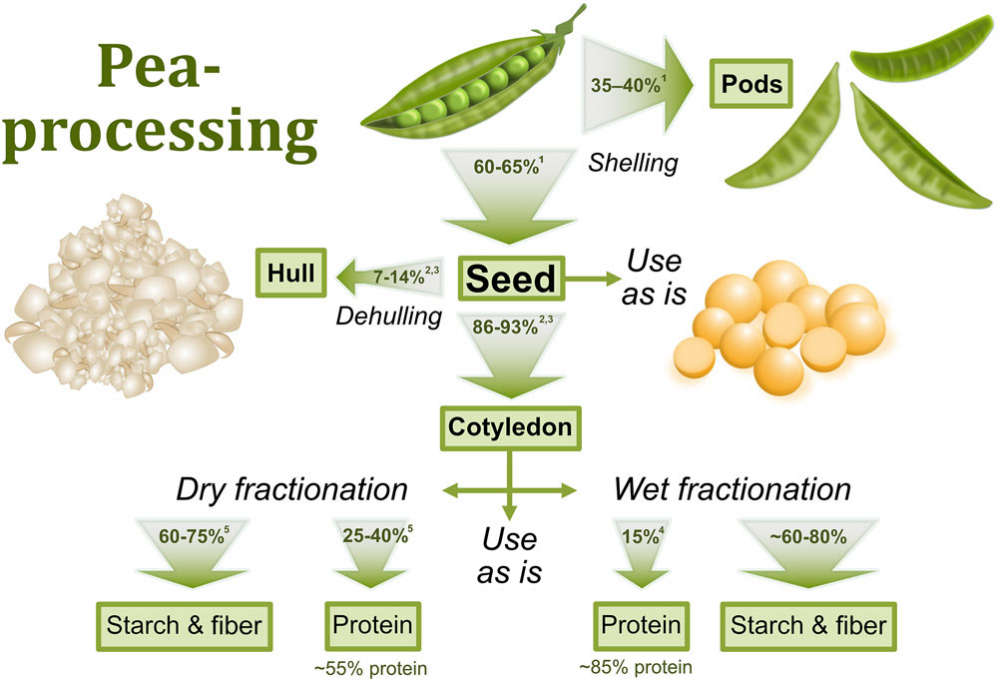
Overview of pea processing for protein‐rich end products and resulting side streams. Percentages within arrows indicate the approximate proportion (by weight) for each stream. ^1^Nasir et al. (2024); ^2^Zhong et al. (2018); ^3^Ali‐Khan (1993); ^4^Feyzi et al. (2018); ^5^Colonna et al. (1980).

Consumption and utilization of peas (*Pisum sativum* L.) and pea‐derived ingredients such as protein isolates have gained increased attention in recent decades (Rogers et al. 2024). The processing of legumes and the extraction of protein typically result in large volumes of side streams rich in starch and fibers. Finding alternative uses for these products, such as functional ingredients in food production, can allow for more efficient use of biomass and increase the economic value from the fractionation of peas and other legumes. Some common fractionation pathways for the up‐concentration of pea protein (and resulting side streams) are shown in Figure 1.

Pods from the pea plant encapsulate the seeds, consisting of the inner kernel cotyledon and the coating hull. The pod comprises approximately 35%–40% of the total pea weight (seed plus pod) and is most often used as animal feed (Fatima et al. 2024; Nasir et al. 2024). The hull accounts for approximately 7%–14% of the total pea seed weight (Ali‐Khan 1993; Zhong et al. 2018). However, the side stream occurring from dehulling can be proportionally larger as the industrial process of dehulling also removes part of the outermost layer of the cotyledon (Susmitha et al. 2022). The pea hull and pods are parts that are often discarded during protein isolation because of their high fiber but low protein content. The approximate composition of pea cotyledon, hull, whole pea, and pods appears in Table 1. The composition of peas and other legumes varies depending on such factors as the anatomical part of the pea, variety, time of harvest, and cultivation conditions (Daveby et al. 1993; Salawu et al. 2001). Because the cotyledon tends to be rich in protein, this section of the pea is suitable for producing protein concentrates and protein isolates. However, the cotyledon is also rich in starch and fiber. Thus, during protein fractionation, these are major side streams suitable as food ingredients.

**TABLE 1:** Proximate composition (g/100 g dry matter) of pea cotyledon, hull, whole seed, and pod.

	Protein (g/100 g)	Starch (g/100 g)	Fiber (g/100 g)	Fat (g/100 g)	Ash (g/100 g)	References
Cotyledon	23–30	31–52	6–9	2–3	2–3	Mondor 2020; Daveby et al. 1993
Hull	4–14	1–12	71–92	0–1	2–3	Dalgetty and Baik 2006; Ramirez et al. 2021; Reichert 1981; Leterme et al. 1996; Ralet et al. 1993
Whole seed	15–25	20–42	15–31	2–5	3–7	Martín‐Cabrejas et al. 2003; Kan et al. 2018
Pod	11–14	4	40–59	1–4	5–7	Mateos‐Aparicio, Redondo‐Cuenca, Villanueva‐Suárez, et al. 2010; Hanan et al. 2020; Mejri et al. 2019

Pea starch and pea fiber can be added for functional properties and to provide nutritional value and health benefits to foods. Pea starch added in the form of resistant starch (RS) can resist digestion in the small intestine and instead be fermented in the colon, potentially providing health benefits to the consumer (Bojarczuk et al. 2022). Consumption of dietary fiber has been associated with a range of health benefits, such as reducing the risk of obesity and certain types of cancer (Y. He, Wang, et al. 2022). For a more detailed discussion on the potential health benefits of consumption of peas and pea‐derived products, as well as starch and fiber in general, the reader is referred to the reviews by T. Kumari and Deka (2021), Wu et al. (2023), Nasir et al. (2024), Bojarczuk et al. (2022), and Y. He, Wang, et al. (2022).

Pea starch and pea fiber can be used in a range of products such as bread, noodles, pasta, meat, new plant‐based formulations, and beverages (Pietrasik et al. 2020; J. H. Li and Vasanthan 2003; Sanz‐Penella et al. 2010; Y. Li et al. 2024). Pea starch is an attractive ingredient for the industry due to its nonallergenic properties and its lower cost compared to corn, wheat, and potato starches (Ratnayake et al. 2002). Pea fiber can also be added to increase nutritional value or as a functional ingredient, but its applicability may be limited by low solubility and poor technofunctional properties (Elleuch et al. 2011; T. Kumari and Deka 2021; T. Kumari et al. 2025). Additionally, the associated undesirable sensory effects of incorporating pea starch or fiber are one of the main reasons for the current underutilization of these components (Tassoni et al. 2020). Hence, finding treatment methods to increase functionality and further understanding the sensory attributes of pea starch and pea fiber are crucial for consumer acceptance and success in their utilization in food production. By linking the sensory attributes to biochemical composition (such as macronutrients and volatile compounds) and physicochemical properties (such as particle size and water‐ and oil‐holding properties), an in‐depth understanding of the gastronomical possibilities of pea starch/fiber‐enriched products can be achieved (Ji et al. 2023; Wu et al. 2023). Altogether, identifying the characteristics of pea starch and fiber can be beneficial for creating new food applications and enhancing future products. This knowledge may be of direct benefit to industrial stakeholders, to the academic community, and/or indirectly to consumers.

While several recent reviews have examined pea protein and its applications in the food industry (Ge et al. 2020; Lam et al. 2018; Z. X. Lu et al. 2020; Mathew et al. 2024), the other main components of peas and side streams generated during pea protein extraction, that is, starch and fiber, have received comparatively little attention. This narrative review addresses this gap by providing a focused synthesis of current research on the functional and sensory properties of pea starch and fiber in food applications. It further investigates how these properties are influenced by various chemical, physical, and enzymatic modification methods, offering new insights into their potential for value‐added use. The outline of this review is organized as follows: First, an overview of the composition and processing of peas is provided. Subsequently, the composition of pea starch and pea fiber is discussed, followed by a summary of modification techniques used to alter functional properties. The work then explores the use of pea starch and pea fiber in different foods and how their addition might affect textural and sensory properties. Finally, the sensory properties are evaluated, and future challenges and opportunities in modifying and utilizing pea starch and pea fibers in food applications are discussed.

## 2 Composition of Pea Starch and Pea Fiber

### 2.1 Composition, Structure, Morphology, and Digestion of Pea Starch

Peas belong to the species *P. sativum* L., which includes two genetically different phenotypes: smooth peas (with a smooth seed surface) and wrinkled peas (with a wrinkled seed surface) (Colonna and Mercier 1984; Ratnayake et al. 2002). The wrinkled phenotype is caused by a mutation at the rugosus locus, influencing various chemical characteristics, including the properties and morphology of the starch (Daba et al. 2025; Morales‐Hernández et al. 2024; Ratnayake et al. 2002; Ren, Yuan, et al. 2021), which will affect their use in various food products.

The two major components of starch are amylose and amylopectin. Amylose is an almost linear molecule mainly consisting of α‐(1→4)‐linked d‐glucopyranosyl residues. Amylopectin is a branched molecule consisting of α‐(1→4)‐linked d‐glucopyranosyl residues (Hoover et al. 2010; Ren, Yuan, et al. 2021), as well as α‐(1→6)‐linked d‐glucopyranosyl residues at the branching points. In smooth peas, the amylose content ranges between 29% and 49%, whereas wrinkled peas have a higher amylose content of 60%–78% (Colonna and Mercier 1984; Daba et al. 2025; Ren, Yuan, et al. 2021). For smooth peas, the average chain length of amylose is 1300–1400 (degrees of polymerization) and amylopectin 17–20, whereas for wrinkled peas, the recorded average chain lengths for amylose and amylopectin are 1100 and 32–45, respectively (Ren, Yuan, et al. 2021).

In nature, starch is found as granules containing alternating amorphous and crystalline lamellae, with amylose and short amylopectin chains in the amorphous regions and double‐helical amylopectin in the crystalline regions. Depending on the crystalline packing, different polymorphic types (A, B, and C) exist (Bertoft 2017; Buléon et al. 1998). A‐type starch, found in cereals, has a tighter packing; B‐type, found in tubers, has a looser packing; and C‐type, commonly found in legumes, consists of B‐type crystals around the hilum and central areas of the granule, with A‐type crystallites in the more peripheral areas (Hoover et al. 2010; Ren, Yuan, et al. 2021). Smooth peas, like most legume starches, are primarily of the C‐type. Interestingly, the wrinkled pea starches consist of the B‐type, attributed to the higher amylose content, which predominantly exists in the amorphous regions (Colonna and Mercier 1984; Morales‐Hernández et al. 2024; Ratnayake et al. 2002; Ren, Yuan, et al. 2021).

Daba et al. (2025) reported clear morphological differences between pea starches, describing smooth peas as having predominantly simple, round granules, while wrinkled peas exhibited mainly compound granules. Earlier studies reported overlapping characteristics: smooth pea starch granules typically range from 2 to 40 µm, mainly oval but also spherical or irregular in shape, and contain two size populations (2–8 and 15–30 µm), along with cracks and some compound granules (Ratnayake et al. 2002; Bertoft et al. 1993). Wrinkled pea starches also showed two size populations, but with generally smaller granules and a higher occurrence of compound granules (Bertoft et al. 1993). Wrinkled pea starch had a lower purity than smooth pea, which could be explained by a higher presence of protein that also caused granular adhesion (Daba et al. 2025).

Starch can also be classified into three groups: rapidly digestible starch (RDS), slowly digestible starch (SDS), and RS based on the rate of glucose release and its absorption in the gastrointestinal tract (Włodarczyk and Śliżewska 2021). RDS and SDS can be hydrolyzed by digestive enzymes, albeit more rapidly for RDS and more slowly for SDS. In contrast, RS is resistant to digestion by enzymes and passes through to the colon, effectively functioning as a fiber within the body. Five different forms of RS exist, corresponding to different structural configurations of the starch and/or how it is embedded within the food product, causing digestive resistance. RS type I is starches that are physically inaccessible, for example, within plant cell walls, whereas type II RS starches are native granules naturally resistant to enzymatic degradation due to their granule structure and composition (Włodarczyk and Śliżewska 2021). RS type III starches are retrograded starches, type IV starches are those chemically modified, for example, by crosslinking, to resist enzymatic digestion, and RS type V starches are amylose–lipid complexes resisting amylase digestion (Włodarczyk and Śliżewska 2021). Depending on the type of RS, food processing can cause sufficient alteration for the RS to become digestible, which diminishes its role as an effective fiber (McWilliams 2012).

The content of the various starch classes differs between peas and other food sources (Z.‐H. Lu et al. 2022). Native pea starch has RDS (16%–23%), SDS (33%–37%), and RS (43%–47%) contents between those of wheat and potato, and a larger RS content (14%–23%) compared to both wheat and potato after cooking (Z.‐H. Lu et al. 2022). The lower digestibility of cooked pea starches is attributed to their relatively high content of amylose and RS starch, making pea starch an attractive ingredient in the production of healthy food products with a lower glycemic index (GI) (Daba et al. 2025; Ren, Yuan, et al. 2021; G. Sun et al. 2023). In terms of RS content between wrinkled and smooth pea starch, the results are conflicting. While Ren, Setia, et al. (2021) found that the RS content was significantly higher in wrinkled pea than in smooth pea, Daba et al. (2025) found that the ratio of RS in wrinkled pea was lower (although nonsignificant) than for smooth pea. Particularly during the first 20 min of in vitro analysis, wrinkled pea starch was more digestible than smooth pea starch due to the more porous and vulnerable granular structure (Daba et al. 2025). For a more detailed discussion of the effect of processing and modification methods on legume starch digestibility, the reader is referred to the review by Jeong et al. (2019).

### 2.2 Monosaccharide Composition of Pea Cotyledon, Hull, and Pod Fiber

The amount of fiber in different parts of the pea (such as cotyledon, hull, and pod) differs significantly, with the highest amount found in the hull and pod and the lowest in the cotyledon (Table 1). Based on monosaccharide analysis, the hull has been reported to contain mainly cellulose and some pectic substances, with low levels of other polysaccharides and lignin (Reichert 1981; Karlsson et al. 2024). The pod contains a comparably larger proportion of neutral, non‐cellulosic polysaccharides and lignin, but with cellulose remaining the largest fraction (Bhiri et al. 2024; Galimova et al. 2021). The monosaccharide composition of pea fiber from the different parts of the legume (cotyledon, hull, and pod) is summarized in Table 2. Pea cotyledon fiber contains mainly arabinose, uronic acids, and glucose, while the pea hull fiber is composed of mostly glucose, with significant amounts of uronic acids, xylose, and arabinose (Table 2). The pea pod fibers are mainly composed of glucose and xylose, with some uronic acids and galactose (Mateos‐Aparicio et al. 2012; Mateos‐Aparicio, Redondo‐Cuenca, and Villanueva‐Suárez 2010).

**TABLE 2:** Monosaccharide composition (% of total monosaccharides) of fiber extracted from pea cotyledon, hull, and pod.

	Ara (%)	Fuc (%)	Gal (%)	Glc (%)	Man (%)	Rha (%)	Xyl (%)	Uronic acids (%)	References
Cotyledon	24–43	0–1	2–9	16–26	2–11	2–6	4–6	19–28	Daveby et al. 1993; Guillon and Champ 2002
Hull	4–9	0–1	1–3	57–71	0–2	1–3	6–19	10–17	Ramirez et al. 2021; Leterme et al. 1996; Ralet et al. 1993; Le Goff et al. 2001; Guillon and Champ 2002; Karlsson et al. 2024
Whole pea	18	n.d.	tr	41	1	tr	5	31	Martín‐Cabrejas et al. 2003
Pod	2–3	0–1	3–6	40–45	0–1	0–1	35–36	13–14	Mateos‐Aparicio et al. 2012; Mateos‐Aparicio, Redondo‐Cuenca, and Villanueva‐Suáre 2010

Abbreviations: Ara, arabinose; Fuc, fucose; Gal, galactose; Glc, glucose; Man, mannose; Rha, rhamnose; n.d., not determined; tr, traces; Xyl, xylose.

As the major fraction of pea fiber is insoluble, the monosaccharide composition of the insoluble fiber is typically similar to that of the total dietary fiber (TDF). On the other hand, the monosaccharide composition of the soluble fraction might differ significantly. The reported monosaccharide composition of soluble fiber extracted from the cotyledon differs between studies, but high levels of uronic acids, arabinose, and galactose are generally reported (Brummer et al. 2015; D. Wu, Wan, et al. 2023; Martín‐Cabrejas et al. 2003). Differences in composition may be related to the different extraction procedures used. In general, soluble fiber extracted from pea cotyledon contains mainly arabinose and galactose and significant amounts of xylose, galacturonic acid, and glucose (Brillouet and Carré 1983). Soluble fiber fractions extracted from pea pods contain high levels of uronic acids (>50%), substantial amounts of galactose (26%), and some arabinose (8%) (Mateos‐Aparicio et al. 2012). The high levels of uronic acids, galactose, and arabinose suggest the presence of galactose‐rich and arabinose‐rich pectin, which are also the main sugars in the suggested pea pectin structure reported by Noguchi and colleagues (Brummer et al. 2015; Noguchi et al. 2020).

## 3 Modifications for Improved Functionality

Multiple modification methods are available, for both starch and fiber, to improve the functionality for different applications. Table 3 summarizes the modification methods for starch and fiber discussed in this review paper, as well as some of the main effects on the starch and fiber.

**TABLE 3:** Summary of chemical, physical, and enzymatic treatments discussed within this review paper and their effect on pea starch and pea fiber.

Modification method		Effect on starch	Effect on fiber	References
Chemical	Acid treatment	Debranching of amylopectin. Production of thin boiling starches.	Hydrolysis of lignin, neutral noncellulosic polysaccharides, and pectin. Reduced hydration properties (SC, WHC, WUC) and increased SDF content.	Gutöhrlein, Drusch, et al. 2020; Gutöhrlein, Morales‐Medina, et al. 2020; McWilliams 2012; Ramirez et al. 2021; Zhang et al. 2019
	Alkaline treatment	Production of thin boiling starches.	Removal of lignin and solubilization of neutral noncellulosic polysaccharides. Increased hydration properties (SC, WHC) and SDF content.	Gutöhrlein, Drusch, et al. 2020; J. H. Li and Vasanthan 2003; McWilliams 2012; Ramirez et al. 2021; Weightman et al. 1995
Physical	Heat treatment	Granular swelling, water absorption, gelatinization, and pasting. Increase viscosity and gel network formation (during cooling).	Particle swelling and increased amount of soluble material. Increased viscosity of fiber dispersions.	Karlsson et al. 2024; Arrigoni et al. 1986
	Pregelatinization (starch)	The resulting starch can be used as a thickener without heating.		McWilliams 2012
	Ultrasound	Molecular chain breakage, starch grain recrystallization, and generation of radicals. Increased content of resistant starch.	More porous particle structure. Increased/unchanged SC and WHC.	Akbas et al. 2025; Kalla‐Bertholdt et al. 2023; T. Kumari et al. 2022a; Mhaske et al. 2024; N. Wang et al. 2022; Z. Wu et al. 2022
	High pressure	Alterations in granule crystallinity and morphology.		Leite et al. 2017; Lin et al. 2024; M. Liu et al. 2018
	Irradiation (electron beam, γ‐irradiation)	Depolymerization of starch while maintaining granular structure. Increased viscosity. Formation of radicals.	Cracks and pores on the particle surface, and increased hydration properties.	C. He et al. 2024; Mhaske et al. 2024; J. Zheng et al. 2023; T. Cheng et al. 2022
	High‐pressure homogenization	Reduced and narrow gelatinization temperature.		Villanova and Lin 2022
	Grinding (ultrafine/superfine grinding, high‐energy media mill, colloidal milling)		Reduced particle size and increased surface area and pore volume. Changes in surface morphology. Increased SDF content and minor changes in hydration properties. Increased viscosity of fiber dispersions.	L. Li et al. 2022; Y. Li et al. 2024; M. Wang et al. 2021
	Microfluidization	Increased granule swelling and pasting viscosity. Reduced crystallinity and gelatinization enthalpy.	Reduced particle size and increased specific surface area. Particle disruption and looser structure. Increased water‐ and oil‐holding capacity.	X. He, Dai, et al. 2022; X. He et al. 2023; Ji et al. 2023; Morales‐Medina et al. 2020
	Extrusion		Reduced particle size and increased SDF content.	Ralet et al. 1993
	Pulsed electric field	Increased amylose content and gelatinization temperature.		Y. Liu et al. 2025; Q. Li, Wu, et al. 2019
Enzymatic	Maltogenic amylase	Reduced amylose/amylopectin chain length. Reduced paste viscosity and reduced retrogradation.		D. Li, Fu, et al. 2021; J. Li, Li, et al. 2021
Enzymatic	Mixture of carbohydrolases		Degradation of polysaccharides and reduction in total dietary fiber. Increased SDF content.	Zink et al. 2024
	Pectinase		Degradation of pectin. Increased SDF content and reduction in total dietary fiber.	Zink et al. 2024
	Cellulase		Degradation of cellulose.	Zink et al. 2024

Abbreviations: SC, swelling capacity; SDF, soluble dietary fiber; WHC, water‐holding capacity; WUC, water uptake capacity.

Most treatment methods can be divided into chemical, physical, or enzymatic approaches. In general, chemical treatments are straightforward to implement and present limited issues concerning scalability. However, concerns may arise regarding the necessity for extensive purification of the sample posttreatment, as well as the handling and disposal of waste with chemical reagents (Gan et al. 2021). The energy cost will depend on the temperature requirements and the extent of the purification process. Physical treatments avoid the use of chemicals and reagents and are typically cost‐effective and scalable (T. Kumari et al. 2025). However, this will depend on the modification method, where processes such as microfluidization may impose practical challenges with fibrous materials, and extrusion and high‐pressure processing may require a high‐energy input. Enzymatic treatments allow highly specific modifications and can be used with mild processing conditions (Gan et al. 2021). Limitations may involve the high cost of enzymes and longer treatment times. For a more general discussion of modification methods of starch and dietary fiber, the reader is referred to the reviews by Gan et al. (2021), B. Kumari and Sit (2023), and T. Kumari et al. (2025).

### 3.1 Modifications and Functionalization of Pea Starch

**FIGURE 2:**
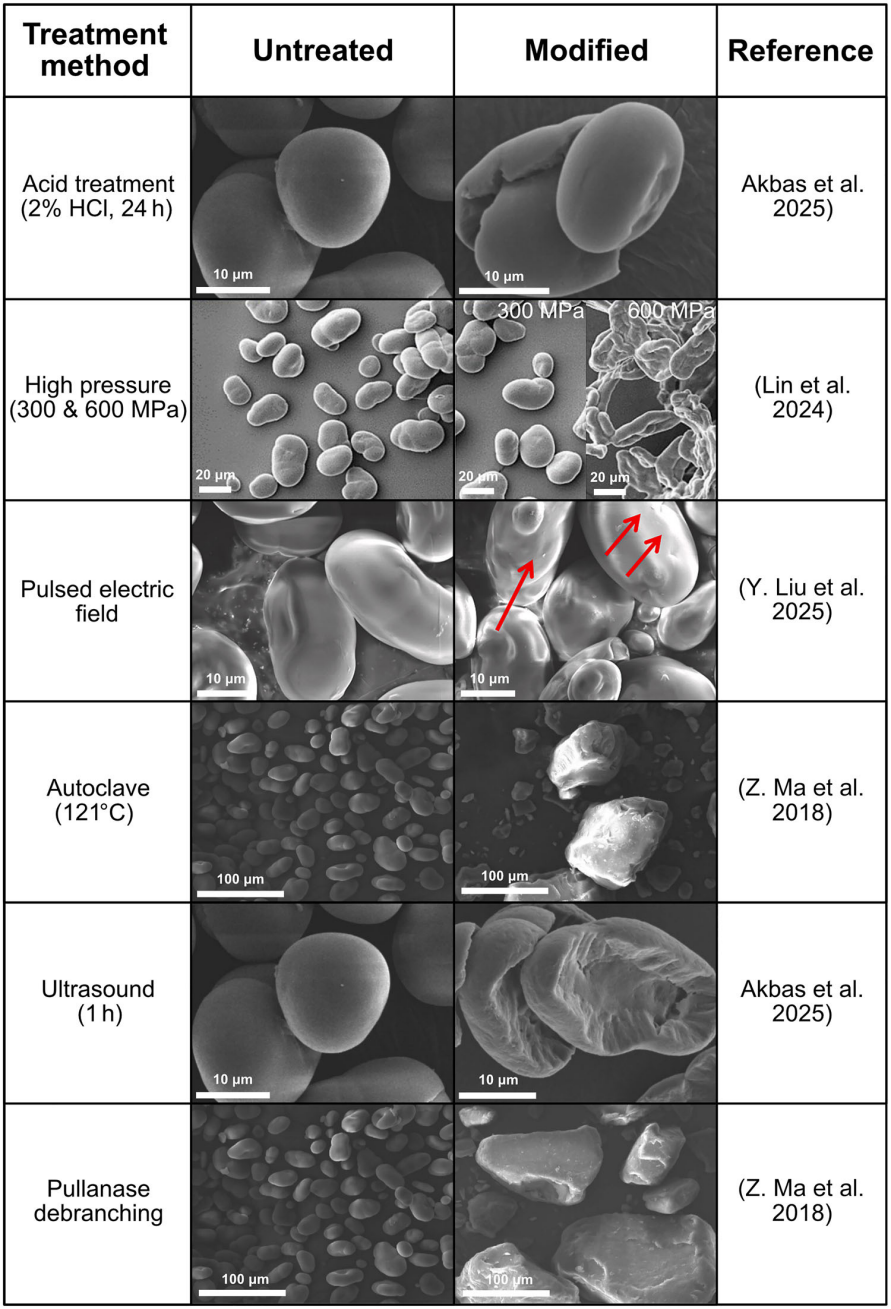
Structural changes of pea starch granules after different treatment methods. Micrographs were obtained from the corresponding references and reproduced with permission from the original publishers. Figures adapted with permission from Elsevier (Lin et al. 2024; Y. Liu et al. 2025; Z. Ma et al. 2018). Figure adapted from Akbas et al. (2025) with permission from Springer and the Creative Commons Attribution (CC BY) license.

Physicochemical factors limiting the use of pea starch in industrial applications include its poor thermal stability, low acid and shear resistance, insolubility in water, syneresis, low pasting viscosity, and a high tendency to retrogradation because of its high amylose content (Morales‐Hernández et al. 2024; Ren, Yuan, et al. 2021). Modification methods involving physical, chemical, or enzymatic treatments (alone or in combination) can be applied to address these limitations and to improve the structural, physical, or chemical properties of pea starch for different applications (Morales‐Hernández et al. 2024). The effect of the different modification methods (such as decreasing digestibility and altering viscosities at different temperatures) influences the suitability of the modified starches in different food products. Figure 2 highlights how different modification techniques can alter the starch granule morphology in different ways, which may impact the functional properties.

Upon heating in food systems, starch granules absorb water, swell, and undergo gelatinization, an irreversible transition from an ordered to a disordered structure (Vamadevan and Bertoft 2015). Smooth pea starch exhibits a narrower gelatinization range (58°C–81°C) than wrinkled pea starch (56°C–112°C), while wrinkled pea starch shows greater thermal stability, attributed to higher amylose content and longer amylopectin branch chains (Ren, Yuan, et al. 2021), consistent with observations in other legumes (Nilsson et al. 2022).

Pea starches generally form stable, high‐viscosity pastes and firm, elastic gels due to their relatively high amylose content, which restricts granule swelling, enhances granule integrity during pasting, and reinforces gel networks during cooling (Wani et al. 2016; Hoover et al. 2010; Morales‐Hernández et al. 2024). This high amylose content also promotes retrogradation and RS formation, contributing to a lower GI compared to cereal starches (Bangar et al. 2022). Differences between pea types have been reported, with smooth pea starches showing greater swelling power and developing pasting curves at lower temperatures (≤95°C), whereas wrinkled pea starches require higher temperatures (up to 140°C) to fully gelatinase and paste, reflecting their higher amylose content and amylose–lipid complex formation (Daba et al. 2025; Cornejo‐Ramírez et al. 2018).

#### 3.1.1 Chemical Treatments

Chemical treatments can modify the properties of starch by partially or entirely destroying the hydrogen bonds within it and inserting new functional groups (Haq et al. 2019; McWilliams 2012). Examples of chemical treatments include acid hydrolysis, oxidation, crosslinking, esterification, and etherification (McWilliams 2012; Morales‐Hernández et al. 2024).

Wet extraction of protein typically involves a solubilization step at alkaline pH (ca. pH 8–10) where the protein can be separated from a starch and fiber‐rich insoluble fraction. It is well known that alkaline treatment may affect the pea starch properties (Q. Sun et al. 2015; S. Wang and Copeland 2012). The effects of 0.1 M NaOH for 15 days have been shown to reduce amylose content and pasting viscosity while increasing in vitro enzymatic breakdown (S. Wang and Copeland 2012). However, most studies investigate treatment conditions harsher than those typically used during combined protein and starch/fiber extraction. Nonetheless, it has been observed for other starches (e.g., maize and rice) that mild alkaline treatment (pH around 8–10) may also affect starch properties (Xu, Liu, Zhang, Ma, et al. 2024; Xu, Liu, Zhang, et al. 2024). For pea starch isolations, comparison between neutral, alkaline, and acid extraction methods has shown limited differences in amylose content but some differences in pasting behavior and gelation properties (Q. Sun et al. 2015). N. Wang et al. (2022) found that by using ultrasound‐assisted alkali extraction methods rather than conventional alkali extraction, the functional properties as well as the extraction yield (increase of 13%) of pea starch were improved. The molecular modifications caused by the ultrasound treatment resulted in increasing the ratio of amylose as well as promoting the formation of RS caused by retrogradation, all properties sought after in the production of low‐calorie starchy foods.

Thin boiling starches can be produced by acid hydrolysis (hydrochloric or nitric acid) or alkali (sodium hypochlorite) treatment. Akbas et al. (2025) found that acid treatment increases the gelatinization temperature and reduces the swelling power and viscosity of pea starch. Zhang et al. (2019) investigated the effect of acid hydrolysis on the physicochemical properties of pea starch and its film‐forming capacity. The acid hydrolysis treatment predominantly affected the amorphous regions. It rendered a more refined crystallinity in the residual starch and improved the orientation of the hydrolyzed starch crystallites, thus augmenting the relative crystallinity. The acid hydrolysis treatments increased the tensile strength of the films, likely because of the formation of the less‐branched and lower‐molecular‐weight molecules. These molecules could function as a slippage area between larger amylopectin molecules during viscous flow, while bringing more chain alignment through hydrogen bonding during the drying process.

Oxidized starches undergo oxidation reactions through alkaline treatments. They are thin‐boiling but form softer gels when cold. This could explain their limited use in the food industry (McWilliams 2012). J. H. Li and Vasanthan (2003) found that hypochlorite oxidation of field pea starch reduced the starch viscosity during pasting and negatively impacted the noodle quality because it also reduced the gel‐forming capacity.

Crosslinking of pea starch by different phosphorylation methods has been shown to increase the amylase resistance and the levels of RS in both gelatinized and ungelatinized starch (Dong and Vasanthan 2020). The increasing effect differed depending on the phosphorylation method used but consistently increased with an increased degree of crosslinking. Esterification of pea starch with malic acid has been shown to significantly reduce the crystallinity and increase the levels of RS (Shi et al. 2019). Etherification by hydroxypropylation of pea starch can reduce the starch pasting viscosity and, when performed on debranched pea starch, create an almost completely amorphous structure by destroying the crystalline regions of the starch (Tang et al. 2018).

#### 3.1.2 Physical Treatments

Pregelatinized starches are starches that have been dehydrated after being cooked, swollen, and gelatinized (McWilliams 2012), either through heat‐moisture treatment (HMT) or annealing. Such starches can be used as thickeners in products without requiring heating. HMT has been shown the ability to increase the gelatinization temperature, reduce the pasting viscosity, and enhance the enzymatic resistance of pea starches (F. Cheng et al. 2024). Annealing pea and potato starch in ethanol solution is a comparatively rapid modification method that improves the heat, shear, and acid stability of starches because the complexes formed between the ethanol and the starch stabilize the helical chains (Chen et al. 2022). The higher tolerance of these starches in different conditions widens their utility as a textural starch in products such as deep‐fried foods and soups.

There are also nonthermal modification treatments. These can provide alternative modification routes, avoiding the use of chemicals, enzymes, and heating. The reviews by Mhaske et al. (2024) and Z. Wu et al. (2022) provide a good, comprehensive overview of the vast subject of nonthermal starch treatments. Nonthermal treatments modifying pea starches include sonication, ultrasound, microfluidization, high hydrostatic pressure, irradiation, and pulsed electric field (PEF).

During ultrasound treatments, sound frequency waves cause cavitation bubbles. These agitate particles in a solution, locally causing extreme temperature and pressure changes and resulting in a degradation of macromolecular C─C bonds and the generation of long‐chain radicals (Akbas et al. 2025; Mhaske et al. 2024; Z. Wu et al. 2022). You et al. (2019) found that ultrasound could increase the RS content of peas, mainly due to the cavitation causing molecular chain breakage and starch grain recrystallization. Hence, this could be a useful treatment method to increase the health benefits of pea starch. Akbas et al. (2025) reported that ultrasound treatment (>45 min) can effectively modify pea starch, producing effects comparable to 24 h acid hydrolysis while being more time‐efficient and environmentally friendly. The treatment increased apparent amylose content and caused granule damage (Figure 2) and reduced crystallinity. These structural changes increased solubility but decreased swelling power, thermal stability, and starch clarity, potentially resulting in denser starch‐based structures.

High‐pressure processing is a nonthermal treatment method that subjects the sample to high pressures under controlled temperature and times. There are conflicting results on how high‐pressure treatments affect the gelatinization properties of pea starch (Leite et al. 2017; Lin et al. 2024; M. Liu et al. 2018). According to Leite et al. (2017), higher pressure treatments (500–600 MPa) for 15 min were required to promote cold gelatinization of pea starch as indicated by an increase in apparent viscosity. Similar pressures have been observed by Lin et al. (2024) to be needed for larger changes in granule structure and gelatinization to occur (Figure 2). Lower pressures (400 MPa and under) did not alter the morphology or structure of the starch granule enough to impact the apparent viscosities (Leite et al. 2017). In contrast, M. Liu et al. (2018) found that pea starch treated for 25 min at 600 MPa exhibited the lowest viscosity value and that the viscosity of pea starch increased with increased pressure between 150 and 450 MPa. The observed difference between the studies could relate to the longer exposure times and higher temperatures used in the study by M. Liu et al. (2018). These may have induced greater alteration in the starch granule's crystalline structure and morphology, which was destroyed after pressure treatments at 600 MPa for 25 min. The observed differences may also be related to potential differences in amylose content in the starches used in the two studies, where a lower amylose content typically results in a lower energy input needed for gelatinization (Leite et al. 2017). Another observation was that starch digestibility decreased with greater applied pressure, as RDS levels dropped and RS levels rose. This was linked to changes in starch crystallinity and increased molecular interactions, which reduced amylolytic enzyme activity (M. Liu et al. 2018).

Microfluidization, a homogenization technique using high pressures to force liquid suspensions through small microchannels, has also been explored for the modification of pea starch (X. He et al. 2023). Microfluidization of pea starch led to granule deformation, reduced crystallinity, and increased swelling, while still conserving the granule integrity (X. He et al. 2023). Simultaneously, the enthalpy of gelatinization decreased, and pasting viscosity increased. Based on the results, microfluidization was suggested as an effective industrial way to produce partially gelatinized pea starch (X. He et al. 2023).

Electron beam irradiation (EBI) can be used to alter the molecular structure and properties of starch by exposing the starch to a beam of high‐energy electrons. Low‐dose EBI treatment (<10 kGy) has been shown to not significantly affect pea starch digestibility while increasing the crystallinity and gelatinization temperature of pea starch and reducing the amylose content, pasting viscosity, and final viscosity (C. He et al. 2024). Furthermore, pre‐treating starch with EBI has been found to facilitate the preparation and enhance the structural and physicochemical properties of starch nanocrystals (J. Zheng et al. 2023), because the EBI causes depolymerization of starch and positively impacts properties such as relative crystallinity, short‐range orderliness index, solubility, apparent amylose content, zeta potential, and flow.

A novel treatment method that has gained increased interest in food processing in recent years is PEF (Arshad et al. 2021). PEF treatment is a nonthermal processing method using short bursts of high‐voltage electricity to permeabilize cell membranes. PEF treatment has been shown to increase the amount of RS in pea starch while reducing the swelling power and storage modulus of the starch paste (Y. Liu et al. 2025). It also resulted in changes in the starch granule morphology, with increased roughness, pores, and damage on the granule surface (Figure 2) (Y. Liu et al. 2025).

A summary of thermal and nonthermal physical modification techniques, and their effect on legume starches in general, is given in the review by Rostamabadi et al. (2024).

#### 3.1.3 Enzymatic Treatments

Enzymatic modification of starch creates new structures by altering molecular mass, branch chain‐length distribution, and the amylose‐to‐amylopectin ratio when enzymes interact with gelatinized starch (Punia Bangar et al. 2022). A general review on the use of enzymes to modify starches is given by Bangar et al. (2022).

Treating pea starch with maltogenic amylase has been reported to reduce the chain length of amylose and amylopectin, causing a reduction in the paste viscosity and impeding retrogradation (D. Li, Fu, et al. 2021; J. Li, Li, et al. 2021). Additionally, 24 h after the enzymatic treatment, the RS content of the cooked pea increased by 4.2%. Even larger increases in RS content have been observed in other studies, where an increase from around 5% RS to 35% RS has been observed (Y. Zheng et al. 2022). The differences between studies might be explained by differences in enzyme activity and treatment conditions. Shi et al. (2014) also found that enzymatic treatment, β‐amylase or a combination of β‐amylase and transglucosidase, augmented the proportion of slowly digested starch. The enzymes shortened the amylopectin chains, reducing the crystallinity and altering branch density and pattern, all of which influenced starch digestibility.

Z. Ma et al. (2018) also transformed the crystalline conformation of starch through a combined enzymatic treatment: autoclavation with α‐amylase and pullulanase de‐branching. The treatment caused the starch to become more compact while also promoting the formation of double helix structures. The cited literature shows that through various enzymatic treatments, pea starch structures can be created that are more resistant to digestion. This may be of interest to the food industry when designing new, functional, healthy food ingredients.

#### 3.1.4 Combined Treatments

A combination of modification methods may also be applied to enhance alterations. Villanova and Lin (2022) applied high‐pressure homogenization in combination with gallic acid to alter starch structure and digestibility. The high‐pressure treatment reduced and narrowed the gelatinization temperatures of the starch, while the addition of gallic acid (5% or 10%) further weakened the interactions between starch molecular chains. Adding gallic acid reduced crystallinity by 35%, increased the complex index, and raised RS levels, resulting in a 23% decrease in digestion rate. The bonding between gallic acid and starch likely disrupts starch interactions, delaying retrogradation, whereas homogenization treatments promote retrogradation by causing molecular leaching.

W. Li, Zhang, et al. (2019) investigated the effect of ultrasonic treatment of pea starch in acid and salt systems. Ultrasonic treatment reduced pea starch viscosity and enhanced its thermal and cold stability in acid and salt systems, affecting viscosity parameters and reducing gel texture. Higher ultrasonic power (0–450 W) led to glycosidic bond breakage, lower crystallinity, and surface damage on starch particles, with more pronounced effects in acid (citric acid, pH 3) than in salt systems (0.5 M NaCl) (W. Li, Zhang, et al. 2019).

Han et al. (2021) investigated the effects of HMT assisted by pre‐ and post‐ultrasound treatment on the physicochemical, structural, and digestive properties of pea starch. The ultrasound treatment of pea starch partially depolymerized amylopectin chains. This resulted in a greater number of linear fragments and an increase in the amylose content from 34% to 38%, plus an increase in the pasting viscosities (Han et al. 2021). Simultaneously, HMT and combination treatments caused a decrease in both amylose content and pasting viscosity. This was likely due to starch chain interaction and interaction between amylose and lipids. All treatments decreased the crystallinity, molecular weight, swelling power, and solubility at 70°C–90°C and elevated the RS content, correlating to the induced structural changes. HMT has also been combined with acid hydrolysis, where higher levels of acid hydrolysis prior to HMT treatment resulted in starches with greater thermal stability (Gonzalez and Wang 2023). The changes were suggested to result from the acid hydrolysis, yielding greater amounts of linear chains, which were then capable of reorganizing into crystallites during HMT (Gonzalez and Wang 2023).

### 3.2 Modifications and Functionalization of Pea Fiber

Fiber‐rich materials, such as side streams from pea protein extraction, can be used as functional ingredients in different foods and beverages. A review on the effect of modification of dietary fiber in general can be found in the paper by T. Kumari et al. (2025). However, poor technofunctional properties, such as low solubility and large particle size, can limit their applicability and use. To address this, chemical, physical, and enzymatic treatments can be used to modify their functionality.

Technofunctional properties also play a role in the potential health impact of fibers, making modifications a potential route for improved health effects (Y. He, Wang, et al. 2022). High values of WHC, SC, OHC, and viscosity have all been correlated to the positive health benefits of consuming fiber (Y. He, Wang, et al. 2022). Furthermore, modifications may also affect physicochemical properties such as glucose and cholesterol adsorption capacity, important for metabolic and cardiovascular health (Y. He, Wang, et al. 2022). Hence, modifications and functionalization of pea fiber may provide not only improved functional properties but also potential improved health benefits.

**FIGURE 3:**
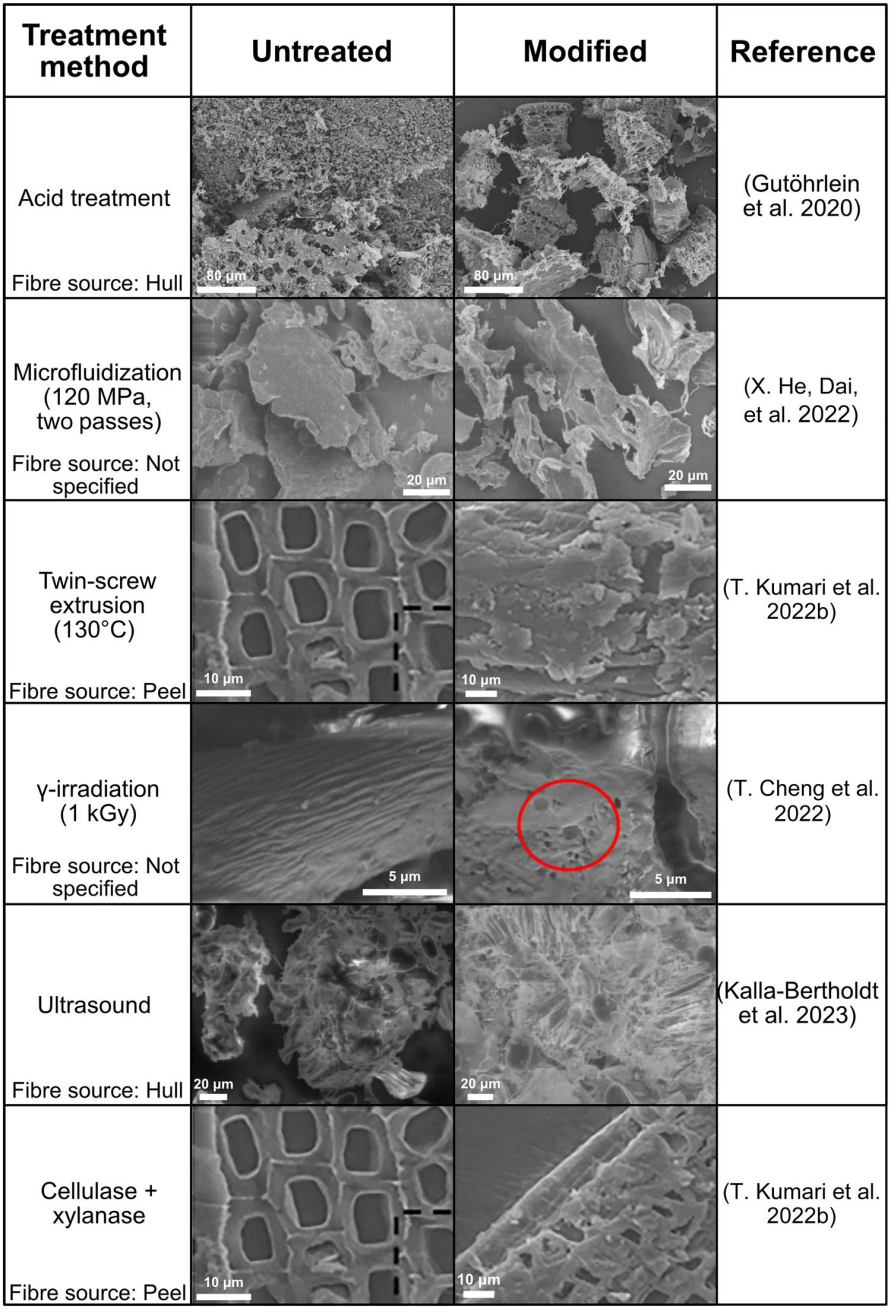
Structural changes of pea fiber after different treatment methods. Micrographs were obtained from the corresponding references and reproduced with permissions from the original publisher. Figure adapted with permission from Elsevier Gutöhrlein, Morales‐Medina, et al. (2020). Figure adapted from T. Kumari et al. (2022b), by permission of Oxford University Press. Figures adapted from X. He, Dai, et al. (2022), Kalla‐Bertholdt et al. (2023), and T. Cheng et al. (2022) with permission from MDPI and the Creative Commons Attribution (CC BY) license.

An overview of studies performed on the functionalization of insoluble pea fibers and the effect on some of their physicochemical properties is given in Table 4, with examples of microstructural changes shown in Figure 3. It should be noted that the methods used to quantify the swelling capacity, water‐holding capacity, and oil‐holding capacity vary, for example, by the use of different centrifugal forces and sample‐to‐liquid (water/oil) ratios. Hence, the values from different studies are not always directly comparable. The development of a standardized evaluation method is hindered by the considerable variability in fiber properties arising from diverse sources and treatments, where the swelling and water/oil adsorption may vary largely.

**TABLE 4:** Summary of studies of the effect of different treatments (physical, chemical, and enzymatic) on the physicochemical properties of insoluble pea fibers. Studies of soluble pea fibers and studies that do not include a clear reference material (control/untreated sample) have been excluded from the table.

Pea fiber origin	Treatment	SC (mL/g)	WHC (g/g)	OHC (g/g)	Particle size, *D*_50_ (µm)	IDF/SDF ratio	Reference
Hull	Control (fine: 50–100 µm)	10.5	7			3.6	Gutöhrlein, Morales‐Medina, et al. 2020
	Water (fine)	6.5	3.3			10.7	
	Ethanol (fine)	7.5	3.8			11.4	
	Hot acid (fine)	11	5			6.2	
	Chelating agent (fine)	16	9.3			5.8	
	Control (coarse: 250–350 µm)	6	3.7			26.7	
	Water (coarse)	6.5	3.7			22.9	
	Ethanol (coarse)	5.5	3.6			23.7	
	Hot acid (coarse)	8	3.8			5.7	
	Chelating agent (coarse)	11	6.9			10.0	
Hull	Control (untreated)	5.2	7.4		375^a^		Weightman et al. 1995
	CDTA	8.3	9.5		315^a^		
	CDTA + HCL	13.5	7.8		285^a^		
	CDTA + HCL + KOH	12.6	9.2		340^a^		
	CDTA + HCL + KOH + KOH	8.9	7.1		280^a^		
	CDTA + HCL + KOH + KOH + KOH	4.9	7.2		220^a^		
	KOH	8.2	5.8		360^a^		
	KOH + KOH	9.4			390^a^		
	KOH + KOH + KOH	9.9			350^a^		
Hull	Control (untreated)		3.3	1		16.1	Arrigoni et al. 1986
	Boiled 60 min		5	1		8.5	
	Autoclave 15–60 min		3.7–4	0.9–1.1		9.5–6.5	
	Extruded		3.3			12.1	
Pod	Control (untreated)	4.5	0.1	2.7			T. Kumari et al. 2022b
	Extruded	4.8–5.8	0.3–1.5	2.7–2.8			
Hull	Control (untreated)	0	4		80	16.6	Kalla‐Bertholdt et al. 2023
	Ultrasound (US)	3	5		150		
Not specified	Control (untreated)	11	5	5.8	76		T. Cheng et al. 2022
	Irradiation dose 0.5–5 kGy	16–19	6.0–8.0	6.4–8	65–74		
Not specified	Control (untreated)		12.5	15			Ji et al. 2023
	High‐pressure homogenization 80–140 MPa		18–22.5	16–20			
Not specified	Control (untreated)	4.4	4.2	3	83		X. He, Dai, et al. 2022
	Pre‐pulverized	9.9	6.7	6.5	64		
	Industry‐scale micro‐fluidization 60–120 MPa	13.6–16.7	7.5–8.7	6.3–7.9	32–43		
Hull	(No control reported)						Morales‐Medina et al. 2020
	Microfluidization: lowest intensity: two runs at 520 bar		13		58		
	Intermediate intensity: two to seven runs at 560–1270 bar		17–18		31–47	6.7	
	Highest intensity: eight runs at 2000 bar		34		26	6.7	
Cotyledon	Control (untreated)	5	7		36		Y. Lu et al. 2023
	Colloid milling	12.5	7.5		33		
Not specified	Control (untreated)	11.5	5.6	1.5	312	55.4	M. Wang et al. 2021
	Ultrafine grinding	12.7	6.1	1.9	57	12.6	
Not specified	Control (untreated)	8.7	5.1	2.2			Y. Li et al. 2024
	High energy media mill 30–90 min	11.8–13.8	5.8–7	4.1–4.8			
Pod	Control (untreated)	4.4	0.2	2.7			T. Kumari, Das, Das, et al. 2024
	Cellulase + xylanase	6.9	3.3	3.2			
Pod	Control (untreated)	4.5	0.1	2.7			T. Kumari et al. 2022b
	Cellulase + xylanase	7.8–8.4	2.8–3.3	3.0–3.3			
Hull	Control (MF)		29		22	3.9	Morales‐Medina et al. 2024
	MF + cellulase (C)		33		26	2.2	
	MF + hemicellulase (H)		35		22	3.2	
	MF + pectinase (P)		32		22	4.3	
	MF + combinations of C, H, and P		32–37		20–25	2.5–4.7	

Abbreviations: CDTA, cyclohexanediamine tetraacetic acid; IDF, insoluble dietary fiber; MF, microfluidization; OHC, oil‐holding capacity; SC, swelling capacity; SDF, soluble dietary fiber; WHC, water‐holding capacity.

^a^ Particle size estimated from interpolation of weight distributions from sieving.

The response to modification treatments may vary significantly depending on the fiber source (Arrigoni et al. 1986; Bourmaud et al. 2018; Kalla‐Bertholdt et al. 2023; Y. Lu et al. 2023). Additionally, factors such as particle size and particle morphology will affect the physicochemical properties (Y. Lu et al. 2023). This implies that the physicochemical properties of fibers extracted from the same crop may vary. As an example, a comparison of the water‐ and oil‐binding properties of commercial and laboratory‐extracted pea fibers has shown that there may be significant differences in their oil absorption and hydration properties (N. Wang and Toews 2011). These variations complicate the comparison of fibers from dissimilar sources, as optimal extraction procedures might differ depending on the raw material. In the case of mechanical treatment, the response is expected to be influenced by the inherent mechanical properties, particle morphology, and microstructure of the fiber. These will differ depending on the botanical origin and the specific part of the crop (such as cotyledon, hull, or pod) from which the fiber is derived (Y. Lu et al. 2023; Bourmaud et al. 2018). Therefore, before a comprehensive understanding of how various treatments affect the physicochemical properties of specific fiber materials, it is essential to study each raw material individually.

#### 3.2.1 Chemical Treatments

Different chemicals and solvents can be used to change the properties of fibers, including by modifying, degrading, or solubilizing different components of the fiber. Chemical treatments can alter the material's solubility, crystallinity, porosity, and interactions with water, affecting the fiber's technofunctional properties such as swelling capacity and viscosity. Common treatments involve alkalis, acids, or chelating agents. Alkaline conditions can be used to remove lignin, dissolve neutral non‐cellulosic polysaccharides, and enhance cellulose accessibility (Kim et al. 2016). Similarly, acid treatments can be used to hydrolyze lignin, non‐cellulosic neutral polysaccharides, and pectin to modify the fiber (Abolore et al. 2024).

Gutöhrlein, Morales‐Medina, et al. (2020) investigated the effect of different chemical treatments (water, ethanol, acidic, and chelating agent) on the hydration properties of pea hull fiber. An increase in hydration properties (SC, WHC, WUC) was observed for disodium ethylenediaminetetraacetate (Na2‐EDTA)‐treated samples (chelating agent), while the other treatments generally resulted in reduced hydration properties (Gutöhrlein, Morales‐Medina, et al. 2020). The increase in hydration properties for the pea hull fiber treated with Na2‐EDTA was hypothesized to be related to sodium carboxylate structures screening the pectin carboxylate anions, preventing cell wall hardening and shrinkage during drying, and resulting in microstructural changes.

Chemical treatment of pea hull fiber using CDTA and acid leads to reduced levels of galactose and anhydrouronic acids (pectin) while increasing WHC and SC (Weightman et al. 1995). Similarly, alkaline treatment (KOH) has been shown to reduce xylose and galactose content in pea hull fiber and result in increased SC and WHC (Weightman et al. 1995). However, if the alkaline treatment followed a prior treatment with cyclohexanediamine tetraacetic acid (CDTA) and HCl, the SC and WHC decreased compared to samples treated only with acid and CDTA. The reduction in SC and WHC when CDTA and acid treatment are combined with subsequent alkali treatment might be due to extensive degradation of the cell wall structure and loss of intracellular spaces, which could previously have held water (Weightman et al. 1995).

Alkaline extraction of fiber from pea pods has been shown to increase the amount of soluble fiber, resulting in a fraction with 73% TDF, of which 17% was soluble dietary fiber (SDF) compared to an SDF content of 1% (81% TDF) obtained using ultrasound treatment (T. Kumari et al. 2022a). Also, nitric acid or citric acid at elevated temperatures, as well as subcritical water extraction, has been used to increase the extraction yield of soluble polysaccharides from pea hulls (yielding up to 10% extracted pectic polysaccharides for nitric/citric acid extraction or an increase in SDF content from 5.6% to 11.8% by subcritical water extraction compared to conventional extraction) (Ramirez et al. 2021; Gutöhrlein, Drusch, et al. 2020).

#### 3.2.2 Physical Treatments

Most studies published on the functionalization of pea fibers have focused on different physical treatments, typically utilizing high temperatures, high pressure, instantaneous pressure reduction, or shear to modify the fiber. Physical treatments are often preferred due to a lower cost and the opportunity to avoid the additional use of chemicals (Gan et al. 2021).

Mechanical treatments such as milling and homogenization are believed to mainly affect the functionality of fiber via changes in particle size, surface area, and porosity (Tejada‐Ortigoza et al. 2016; Y. Lu et al. 2023). Multiple grinding and milling techniques for modifying pea fiber have been studied, generally leading to reduced particle size and increased surface area and pore volume (L. Li et al. 2022; Y. Li et al. 2024; M. Wang et al. 2021). Ultrafine grinding can largely reduce the particle size of pea fiber while resulting in a relatively minor increase in WHC, SC, and OHC (M. Wang et al. 2021). Simultaneously, the content of SDF was increased from 1.3% to 5.0% (M. Wang et al. 2021). Similarly, high‐energy media mill (HEMM) treatment can increase the SC, WHC, and OHC of pea fiber and increase the viscosity when mixed with water, as compared to untreated fiber (Y. Li et al. 2024). Increased HEMM duration further increased the WHC, while the OHC was highest at 30 min (the shortest time investigated). Longer treatment resulted in a reduced OHC while remaining higher than for the untreated reference (Y. Li et al. 2024). In contrast, Y. Lu et al. (2023) observed little to no effect of colloidal milling on the particle size of pea fiber, and the general morphology of pea fiber was not significantly changed.

The effect of superfine grinding on soluble pea fiber and the extraction of soluble fiber from pea (cot/hull not specified) has also been investigated (Yang et al. 2024). Superfine grinding increased the water solubility capacity (WSC) and water‐holding capacity, while the oil‐holding capacity of the soluble pea fiber decreased (Yang et al. 2024).

Ultrasound‐assisted extraction of fiber from pea pods resulted in low amounts of SDF (1%) (T. Kumari, Das, and Deka 2022a). The extracted fiber had a more porous structure compared to that of alkaline‐extracted fiber, but with a higher yield of insoluble dietary fiber (IDF) (T. Kumari et al. 2022a). Nonetheless, they showed similar WHC but with a higher SC observed for the more porous ultrasound‐treated fiber (T. Kumari et al. 2022a). Kalla‐Bertholdt et al. (2023) also investigated the effect of ultrasound treatment on pea fiber, and only minor changes in SDF/IDF content were observed. Surprisingly, the results showed an increase in particle size upon ultrasound treatment, possibly due to particle swelling or aggregation. The SC and WHC also increased, which was attributed to increased porosity and opening of the fiber matrix due to cavitation effects (Figure 3) (Kalla‐Bertholdt et al. 2023).

Heat treatment (95°C, 30 min) of pea hull fiber dispersions has been shown to increase the particle size and amount of soluble material (but with little practical significance due to the low overall levels with an increase from 2% to 3%) (Karlsson et al. 2024). Further, the viscosity and storage modulus increased with heating, possibly related to the swelling of the fiber particles (Karlsson et al. 2024). More intense heat treatments such as boiling, autoclaving, and extrusion of pea hulls have also been investigated (Arrigoni et al. 1986). All treatments increased the SDF content, with autoclaving showing the highest and extrusion the lowest increase. Both boiling and autoclaving increased the WHC, while it was unaffected by extrusion (Arrigoni et al. 1986). Extrusion has also been used to modify pea pod fibers, leading to increased WHC and SC (T. Kumari et al. 2022b).

Various types of high‐pressure and high‐shear microfluidization techniques utilizing high pressure and high shear have been shown, in multiple studies, to reduce the particle size and increase the specific surface area of pea fibers (X. He, Dai, et al. 2022; Ji et al. 2023; Morales‐Medina et al. 2020). The effect was more pronounced as the pressure or number of runs increased (X. He, Dai, et al. 2022; Ji et al. 2023; Morales‐Medina et al. 2020). The microstructure is also affected during this process: the particles are broken into pieces, and a looser structure is observed (Figure 3) (Ji et al. 2023; X. He, Dai, et al. 2022; Morales‐Medina et al. 2020; Morales‐Medina et al. 2024). Furthermore, microfluidization may reduce crystallinity and zeta potential, possibly due to structural disruption (X. He, Dai, et al. 2022; Ji et al. 2023). The SC, WHC, and OHC increase compared to untreated samples and continue to increase with increasing intensity (Ji et al. 2023; X. He, Dai, et al. 2022; Morales‐Medina et al. 2020; Morales‐Medina et al. 2024). An increase in WHC, almost 10 times that of nontreated pea hull fiber, has been observed (3 and 29 g water/g of insoluble mass for the control and microfluidized samples, respectively) (Morales‐Medina et al. 2024). However, at a certain intensity, the effect tends to level off and sometimes decrease again as the intensity is further increased (X. He, Dai, et al. 2022; Ji et al. 2023). It has been suggested that intense size reduction can also break micropores and microcapillaries, leading to substantial damage to the fiber structure and a subsequent loss of functionality (Jacobs et al. 2015). Microfluidization intensity had a limited effect on the IDF and SDF content of pea hull fiber (Morales‐Medina et al. 2020). The viscosity and storage modulus increased for dispersions with smaller particle sizes and more disrupted fiber particles (in other words, higher treatment intensity), and the changes in physicochemical properties correlated well with changes in particle size (Morales‐Medina et al. 2020).

γ‐Irradiation is another technique that can be used to modify fibers (T. Cheng et al. 2022). Cracks and pores appear on the particle surface upon irradiation (Figure 3), crystallinity decreases, and the contents of cellulose, neutral non‐cellulosic polysaccharides, and lignin are reduced, possibly due to partial depolymerization into smaller molecules (T. Cheng et al. 2022). The γ‐irradiation has been shown to increase the OHC, WHC, and SC of pea fiber (T. Cheng et al. 2022). This effect depends on the irradiation intensity and initially increases with increasing intensity but starts to decrease again as the intensity is further increased (T. Cheng et al. 2022). A similar trend is observed for the volumetric mean particle size (which initially decreases) and the specific surface area (which initially increases) with irradiation intensity, but changes to the opposite as the intensity is further increased. However, the observed change in particle size was relatively small (<15% reduction in mean particle size) compared to changes observed by, say, microfluidization.

Extrusion cooking of pea hull fiber decreases the particle size and increases the amount of soluble fiber as the specific mechanical energy input is increased (Ralet et al. 1993). Despite the changes in particle size, morphology (Figure 3), and solubility, little to no effect was observed on the WHC and swelling capacity of the extrusion‐treated fiber (Ralet et al. 1993).

#### 3.2.3 Enzymatic Treatments

Enzymatic treatments include using enzymes or combinations of enzymes to degrade or modify specific fiber components. Depending on the enzyme treatment, this could impact the fiber properties differently, including changes in rigidity and potential loosening of the fiber matrix, thereby affecting the technofunctional properties such as swelling capacity and ability to absorb water and oil. A limited number of studies are available on the effect of enzymatic treatments on the physicochemical properties of pea fibers. Zink et al. (2024) used enzyme blends (Viscozyme L [a mixture of carbohydrases], Pectinex Ultra SPL [mixture of mainly pectinases], and Celluclast 1.5L [cellulase blend]) to modify pea fibers in pea protein concentrates. Both Viscozyme and Pectinex increased the amount of soluble fibers but simultaneously almost halved the insoluble fiber content (Zink et al. 2024). Celluclast had a limited effect on the IDF and SDF contents, likely related to the low amounts of cellulose in the protein concentrate (Zink et al. 2024). All enzymes investigated reduced the average molecular weight of the SDF (Zink et al. 2024). T. Kumari, Das, Das, et al. (2024) used a combination of cellulase and xylanase to modify the properties of fiber extracted from pea pods. The enzymatic modification improved the SC, WHC, and OHC. The most significant change was observed for the WHC, increasing from 0.2 to 3.3 g/g (T. Kumari, Das, Das, et al. 2024). The changes in functional properties were suggested to be a consequence of changes in microstructure, with enzyme treatment leading to a more irregular structure and increased surface area (Figure 3) (T. Kumari et al. 2022b; T. Kumari, Das, Das, et al. 2024).

#### 3.2.4 Combined Treatments

Superfine grinding combined with a mixture of cellulase and xylanase has been shown to increase the extraction yield of soluble fiber from pea (cot/hull not specified), increasing from 4.5% to 16.2% of TDF in the starting material (Yang et al. 2024). The treated fiber had a smaller particle size and increased the WSC and WHC, while the oil‐holding capacity remained the same compared to untreated and single‐treatment samples (Yang et al. 2024).

Microfluidization has also been combined with enzymatic treatment of pea hull fiber (Morales‐Medina et al. 2024). Different enzymatic treatments (cellulase, hemicellulase, and pectinase, alone and in combination) were used to partly hydrolyze the cell wall fibers before microfluidization. Samples pretreated with cellulase (alone or in combination with other enzymes) were most efficient in reducing the particle size of the microfluidized samples and releasing high‐molecular‐weight soluble dietary fiber (HMWSDF). The water‐holding capacity increased for all enzyme‐treated samples compared to the microfluidized reference sample (WHC varied between 29 g/g for the control and 32–37 g/g for the enzyme‐treated samples). The viscosity of suspensions from the differently treated fibers was highest for the samples treated with cellulase together with hemicellulase or a combination of all three enzymes. The increase was related to the high content of HMWSDF, high WHC, and the size and morphology of the insoluble particles. On the other hand, the cellulase‐treated sample showed the lowest viscosity, largely related to the extensive degradation of the fiber material to low‐molecular‐weight SDF, disaccharides, and monosaccharides.

Combined ultrasound and alkaline extraction resulted in pea pod fiber with a similar porous microstructure to the ultrasound treatment and a high level of soluble fiber, as with the alkaline treatment (T. Kumari et al. 2022a). The WHC and OHC were improved compared to the individual treatments, while the SC showed values in between the two (T. Kumari et al. 2022a).

In general, among the abovementioned chemical, enzymatic, and physical treatments, the largest effects on particle size and functional properties such as WHC, SC, and OHC tend to be observed from microfluidization and grinding, with the potential for further increases if combined with enzymatic treatments. However, it should be noted that most studies on pea fiber and functionalization by chemical, enzymatic, or physical treatments are made on pea hull fiber. The effect of different treatments might differ if fiber extracted from the cotyledon or pod is used.

## 4 Gastronomical Applications and Sensory Perspectives

### 4.1 Utilization of Pea Starch in Different Food Applications

The ability of pea starch to provide structure and texture to food products, as well as its status as a healthy starch with a low GI, makes it a valuable ingredient that can be incorporated into a range of products. To highlight the wide range of possibilities for using pea starch, this section gives a few examples, from different product categories, where pea starch has been incorporated.

#### 4.1.1 Bakery Goods

Due to its comparatively high levels of RS and amylose, the GI value of bakery goods can be lowered by incorporating pea starch (Krause, Debon, et al. 2022; Krause, Asamoah, et al. 2022; Sanz‐Penella et al. 2010). However, the high amylose content may also introduce formulation challenges related to dough rheology and staling/retrogradation. These may be addressed by changes in the formulation or by different starch modification methods, such as enzymatic treatments discussed in the earlier sections. Sanz‐Penella et al. (2010) reported that bread with satisfactory sensory properties could be produced with flour replacement levels of up to 20% of thermally modified, RS‐rich pea starch. Adding pea starch to the bread increased the dough's water‐absorption levels and extended the development time. With increasing replacement levels of starch, a lower loaf volume and a denser crumb structure with increased hardness and chewiness were observed. This was hypothesized to relate to the decreased gluten content, plus the pea components’ higher water absorption ability, limiting and weakening the gluten network (Sanz‐Penella et al. 2010).

Odor‐active compounds, such as oxidation‐derived volatiles found in pea flour, are associated with unpleasant, green‐beany off‐notes in bakery products (Krause, Asamoah, et al. 2022). This can pose significant problems in product development while using legume‐derived ingredients. By using purified pea starch and pea protein instead of pea flour, the number of volatiles detected was reduced by 100 times for pea protein and 1000 times for pea starch products (Krause, Asamoah, et al. 2022), demonstrating promising results for the inclusion of pea starch while limiting off‐flavors. From a volatiles perspective, maize starch and pea starch were close to identical, with very low detectable volatile levels. This was ascribed to the absence of fat in both starches. The fractionated ingredients were also more prone to the Maillard reaction and caramelization during baking, which can contribute to favorable malty and roasted notes. The more desirable volatiles associated with fractionated ingredients may help justify the use of individual components (and not pea flour) to replace wheat flour.

In another study, the digestibility of sponge cakes with different formulations of wheat starch, pea flour, and pea protein + pea starch/maize was evaluated using the INFOGEST method (Brodkorb et al. 2019; Krause, Debon, et al. 2022). The cakes containing pea starch (in either flour or pure form) had a lower GI. This could be attributed to the elevated amylose and RS content and could be an attractive property in the production of a low‐calorie food alternative. The pea starch granules also showed greater structural stability, further indicating a higher degree of resistance to enzymatic degradation. Scanning electron microscopy images of the different cake crumbs revealed that the pea cakes formed larger, more continuous networks of protein compared to the wheat and maize varieties. In terms of protein digestibility, no difference was observed between the cake containing purified pea proteins and that containing pea flour.

#### 4.1.2 Extruded Products

Extrusion is a process whereby raw materials are forced through a cylindrical barrel to mix, shape, and sometimes cook a product (noodles, breakfast cereals, snacks, and meat analogs). Product parameters that can change during extrusion processing are starch gelatinization and denaturation of proteinaceous material to produce extruded products with new textures (Ajita 2018).

The properties of pea starch, such as its amylose content, granule morphology, thermal properties, and water absorption characteristics, significantly influence its behavior during extrusion (Logié et al. 2018). The high amylose content and crystallinity of pea starch contribute to a reduction in granular expansion and increased resistance to shear degradation, necessitating a higher extrusion temperature to fully melt pea starch (120°C) compared to potato starch (105°C) (Logié et al. 2018). Having the starch in a homogenous molten state before it is shaped by flowing through the die is beneficial to the final texture of the extruded starchy product (Logié et al. 2018; N. Wang et al. 2012; Ajita 2018). The high amylose content of pea starch also affects the texture and firmness of the extrudate, as amylose molecules tend to reassociate upon cooling, leading to a firmer product (J. H. Li and Vasanthan 2003).

N. Wang et al. (2012) investigated how different processing parameters during extrusion influence the production, texture, and cooking properties of pea starch noodles and showed that the pea starch noodles produced under the established optimal conditions were similar in color and cooking time to commercial mung bean noodles but with a firmer texture. Ciardullo et al. (2019) used extrusion to complex the amylose‐rich pea starch with lipids (palmitic acid or mystic acid) to produce functional food ingredients with a high RS content. The complexed starch had a higher RS content when combined with palmitic acid, likely because of the formation of a tighter amylose helical structure. Exposing the starch to more shear (by having a longer residence time in the extruder, for example) caused more molecular damage. This also promoted the formation of amylose–lipid complexes, thereby further improving the RS content (Ciardullo et al. 2019). However, from a texture viewpoint, a tighter molecular structure and a greater proportion of degraded starch may not be ideal, as the extrudate may have a lower final viscosity and less granular expansion.

Consistent with the role of amylose reassociation and extrusion conditions in determining pea starch texture and firmness (J. H. Li and Vasanthan 2003; N. Wang et al. 2012), extrusion combined with high‐temperature drying (F. Cheng et al. 2025) was developed as a scalable, clean‐label alternative to HMT, producing similar structural modifications (F. Cheng et al. 2024). Pea starch was extruded at 37.5% moisture under a low‐temperature profile (≤65°C) and then rapidly heated at 130°C for 1 h, enabling shear‐induced disruption followed by controlled chain reorganization. The combined effects of extrusion‐induced shear and subsequent thermal treatment disrupted granule integrity, altered crystalline organization, and reduced relative crystallinity, while promoting molecular rearrangement and structural entanglements between amylose and amylopectin chains. These restricted chain mobilities resulted in increased gelatinization temperatures, decreased gelatinization enthalpy, and reduced pasting viscosity and gel hardness, consistent with a denser, more ordered starch network. Such extrusion‐driven entanglements also enhanced enzymatic resistance, leading to a 22% reduction in postprandial plasma glucose response compared with native pea starch.

#### 4.1.3 Meat Products and Meat Analogues

Additional starch is usually added to meat analogs and meat‐based products to serve as a filler ingredient with a reduced environmental footprint or as a functional ingredient (Bühler et al. 2022). The starch used in plant‐based meat‐replacing products is often modified for improved functionality compared to its native counterpart (Dobson et al. 2022; Bühler et al. 2022).

Extrusion cooking is one of the most used techniques to create fibrous textures of plant‐based materials mimicking meat texture (Kulikova et al. 2024; Bühler et al. 2022). The study by Jebalia et al. (2019) produced fibrous plant‐based products using pea flour or a blend of pea starch and pea protein. Compared to the products with pea flour, the products consisting of pea starch–protein blend had a more desirable fibrous and more elastic texture. This was due to larger, more aligned protein domains with better interfacial adhesion between the components (Jebalia et al. 2019).

Pietrasik et al. (2020) investigated the effectiveness of replacing wheat crumbs in beef burgers with different ratio combinations of pea starch and pea fiber. By incorporating pea starch, pea fiber, or mixtures of the two, the beef burgers’ hardness, cohesiveness, and chewiness were increased. Higher levels of fiber inclusion also corresponded to a firmer burger texture. There was no significant difference between any of the pea starch–pea fiber blends and the wheat crumb control in terms of sensory analysis and consumer acceptance. This indicates that pea fractions can be used as a gluten‐free binder in beef burgers (Pietrasik et al. 2020).

Pea starch and fiber were used as fat substitutes in low‐fat breakfast sausages, with starch serving as a key binder to reduce cook loss, expressible moisture, and purge loss (Pietrasik and Soladoye 2021a). While this effect was stronger with modified maize starch, pea starch had a greater influence on chewiness and cohesiveness. Although different starches can affect sausage color, consumer and instrumental color assessments showed minimal differences, with all starch‐containing formulations receiving higher acceptability scores than the control. Overall, pea starch had little effect on consumer acceptability but offered functional and cost benefits compared to sausages with no starch.

### 4.2 Utilization of Pea Fiber in Different Food Applications

Multiple studies have explored the effect of adding pea fiber to foods or food model systems such as bread, meat, and beverages. Pea fiber can be used as a functional ingredient to modify a product's physicochemical properties, such as water holding and texture, or to increase the nutritional value by increasing the product's dietary fiber content. This section does not intend to cover all literature and potential uses of pea fiber in different foods but will highlight some examples and opportunities, plus changes in physicochemical and textural properties upon adding fibers to these foods.

Studies on the effect of pea fiber from the hull and cotyledon (soluble and insoluble) on wheat‐based dough and bread based on wheat have shown that the addition of fiber can have different effects depending on the fiber source (Rosell et al. 2006; Dalgetty and Baik 2006). In general, the addition of (1–10 g/100 g flour) pea hull fiber and insoluble cotyledon fiber increases the dough's water absorption, while adding (1–5 g/100 g flour) soluble cotyledon fiber led to a decrease (Dalgetty and Baik 2006; Rosell et al. 2006; J. Wang et al. 2002). The loaf volume tends to decrease irrespective of the fiber source (Dalgetty and Baik 2006). The impact of fiber extracted from pea pods on the textural properties of dough and bread from wheat flour has also been investigated (Fendri et al. 2016). Based on texture profile analysis, the dough hardness, adhesiveness, cohesiveness, and springiness decreased when fiber was added at 0.25–0.5 g per 100 g flour but increased with 0.75–1 g fiber per 100 g flour (Fendri et al. 2016). A similar effect was observed when fiber was added to bread, where a decrease in bread hardness was observed for all fiber additions, albeit more pronounced at the lower concentrations (≤0.75 g/100 g flour) (Fendri et al. 2016).

Extruded pea hull fiber has been successfully incorporated into wheat bread by replacing up to 15% of the wheat flour, without compromising bread quality (Klava et al. 2024). However, higher replacement levels resulted in negative effects on both physical and sensory attributes, including a decrease in specific volume and porosity, as well as a significant pea off‐flavor. Further studies are needed to explore fiber modification methods that may allow for higher incorporation levels in bread and other foods.

Pea fibers have also been used in beef and chicken burgers (Pietrasik et al. 2020; Huber et al. 2016). The addition of fiber to beef burgers reduced weight loss during cooking (Pietrasik et al. 2020), and adding it (in combination with other fibers) as an animal fat substitute in chicken burgers showed good acceptability in sensory tests and similar textural properties to the control with no fiber added (Huber et al. 2016). During high‐moisture extrusion to produce meat analogs, incorporation of pea fibers can be used to improve textural properties and fiber formation by acting as nucleation sites during fracture, leading to a meat‐like fibrous texture (Guan et al. 2026).

Pea fiber can also be added to liquids and dispersions. Challenges when including pea fibers in liquid food formulations typically relate to poor solubility and large particle size, causing a gritty mouthfeel. Both these issues may be reduced by different modification methods, for example, by reducing particle size, as discussed in the previous chapter on modification methods. T. Kumari, Das, and Deka (2024) compared yoghurt with the addition of unmodified and enzymatically treated pea pod fiber. The addition of fiber showed the potential to reduce gel firmness and syneresis. However, little to no difference was observed for most textural and rheological properties between the modified and unmodified fiber when added at the same concentrations.

A more novel technique to reduce grittiness could also be to coat the fiber particles in a protein gel (D'Oria et al. 2024). Pea fibers have also been used to stabilize pea protein beverages, where it has been shown to increase the stability index, highlighting the potential to also use pea fiber in liquid foods (Y. Li et al. 2024). Preprocessing of the pea fiber using an HEMM treatment further increased the stability of pea protein beverages (Y. Li et al. 2024).

A more novel use of pea fiber includes incorporation in inks for food 3D printing. Venkatachalam et al. (2023) investigated the effect of varying macronutrient composition on the printability of pea‐based inks (Venkatachalam et al. 2023). The printability varied with composition, and they concluded that pea fiber was a prerequisite for good printability of inks based on pea protein, starch, and fiber (Venkatachalam et al. 2023). However, it should be noted that the effect of incorporating fiber or fiber‐rich materials in inks for 3D printing will depend on the fiber properties, such as WHC and particle size distribution.

### 4.3 Sensory Properties of Pea Starch and Fiber

Pea starch and pea fiber are, to date, understudied, and little is known about their sensory profiles. However, several studies have explored the sensory properties of these components (and whole peas) when added in small amounts to food matrices. From the available literature, a recent systematic review developed a lexicon of descriptive sensory terms for peas (Lara and Tsiami 2024). From the articles screened, 205 descriptive sensory attributes were identified. The most frequently reported attributes were earthy, floral, green/green peas, nutty, and roasted odor; bean (different wordings), earthy, fatty, grass (different wordings), green/green peas, metallic, and nutty flavor; bitter and sweet taste; and juiciness/moistness and mealy/mealiness texture. The perceived aroma may be attributed to various volatile compounds in the peas. For instance, “beany” may arise from 3‐isobutyl‐2‐methoxypyrazine (IBMP), “floral” from 2‐decanone, grassy from hexanal, “nutty” from 2,3‐dimethylpyrazine, “roasted” from furaneol/acetylpyridine, and “fruity” from various aldehydes and ketones.

The diversity in the sensory profiles of peas can be caused by different factors. Westling et al. (2019, 2024) report that sensory variation between landrace peas is mainly attributed to the accession (variety/cultivar), while location and harvest year did not influence sensory properties in their study. However, landrace peas may also be influenced by light conditions, temperature, and soil. Moreover, the treatment and preparation (such as steaming, roasting, boiling, or freezing) of the peas before analysis are crucial to their perceived sensory attributes (Lara and Tsiami 2024). Hence, it may also be assumed that different fractionation methods contribute to the differing sensory profiles of the resulting pea product.

Among the limited studies reporting on the sensory properties of pea starch and pea fiber, the available research tends to have focused more on the fiber than the starch. Table 5 gives an overview of sensory attributes and/or consumer acceptance of whole peas and products enriched with pea fiber and pea starch. The addition of pea fiber at an inclusion level of 0.5%–6% in animal‐based products (meatballs, burgers/patties, chicken nuggets, and sausages) does not seem to affect the overall liking of the product (Kehlet et al. 2017; Polizer‐Rocha et al. 2020; Polizer et al. 2015; Pietrasik et al. 2020; Anderson and Berry 2000; Besbes et al. 2008; Pietrasik and Janz 2010; Pietrasik and Soladoye 2021a). While pea fiber inclusion in this range does not often exert a perceived impact on sensory attributes, in other cases it adds more granular/gritty, crumbly, starchy mouth‐coating, chalky and firm, less juicy textures to the products. Adding pea fiber to these types of products has the potential to reduce the meat and fat content, which these studies argue to be healthier and more sustainable. Wheat bread enriched with pea fiber was accepted by consumers and had an enhanced shelf life. Wheat bread with added pea starch was accepted up to an inclusion level of 20% (Gómez et al. 2003; J. Wang et al. 2002). Depending on the extraction method of the side streams, pea starch may influence the perception of the final product (Pietrasik et al. 2012) but can help enhance the sensory properties in the right formulation (Pietrasik and Janz 2010; Pietrasik and Soladoye 2021a). Pea starch can be perceived as chalky in certain formulations (such as beverages), with the perceived chalkiness depending on the particle size and inclusion level of starch (K. K. Ma et al. 2024). The literature further described that edible coatings may be improved by adding pea starch, improving smoothness, taste, and overall acceptance of the formulation (Mehyar et al. 2012). Meat‐free sausages, meatballs, and burgers include pea fiber and sometimes pea starch to enhance the sensorial properties of these products (Rasskazova and Kirse‐Ozolina 2020).

**TABLE 5:** Overview of sensory attributes and/or consumer acceptance of whole pea and products enriched with pea fiber and pea starch.

Pea fraction	Product	Sensory attributes	Sensory analysis method	Reference
Pea in general	Raw material (different treatments)	Odor: earthy, floral, green/green peas, nutty, and roasted Flavor: bean (different variations), earthy, fatty, grass (different variations), green/green peas, metallic, and nutty Taste: bitter and sweet taste Texture: juiciness/moistness and mealy/mealiness	Different methods as summarized in the review article	Lara and Tsiami 2024
Pea hull fiber (Fibradan 20, DLG Ingredients, Denmark)^a^	Meatballs with 3.0–6.0 g fiber per 100 g final product	An increase in pea fiber content, compared to the control product, resulted in a texture that was more gritty, crumbly, and firm and less juicy. Pea fiber addition had only a minor influence on odor and flavor.	Descriptive sensory study with a trained panel (*n* = 9) using a continuous 150 mm linear intensity scale (with 1‐cm endpoints).	Kehlet et al. 2017
Pea fiber^b^	Beef with 1% pea fiber (either reduced meat or fat content)	No observed difference in liking of aroma, texture, flavor, or overall acceptability between the control, fat‐reduced, and meat‐reduced beef.	Consumer (*n* = 6) affective acceptance test using a 9‐point hedonic scale.	Polizer‐Rocha et al. 2020
Pea fiber (Pea Fiber I50M—Roquette Freres, France)^b^	Chicken nugget with 2% pea fiber (either reduced meat or fat content)	No observed difference in liking of aroma, texture, flavor, or overall acceptability between the control, fat‐reduced, and meat‐reduced chicken nuggets.	Consumer (*n* = 60) affective acceptance test using a 9‐point hedonic scale.	Polizer et al. 2015
Pea fiber, pea starch, or combinations	Beef burger with 5% of pea fiber, pea starch, or a combination (75:25, 50:50, 25:75)	All blends were accepted by consumers, but a 50:50 ratio was most preferred, according to consumer feedback. Pea fiber tended to affect the sensory attributes, while pea starch had only a minor impact. Pea fiber increases the “crumbly,” “granular/gritty,” “starchy mouth‐coating,” and “chalky” textures.	Consumer tests (*n* = 122): affective acceptance using a 9‐point hedonic scale for flavor and texture. Seven‐point *just about right* (JAR) scales for flavor, firmness, and juiciness and *check all that apply* (CATA) for perception of flavor and texture.	Pietrasik et al. 2020
Pea cotyledon fiber	Ground beef patties with 1.6% pea fiber in different formulations (reduced fat content)	Inclusion of inner pea fiber in beef patties with reduced fat improved tenderness, without compromising juiciness and flavor.	Descriptive sensory test with trained analytical sensory panel (*n* = 10) using 8‐point structured scales.	Anderson and Berry 2000
Pea fiber concentrate (F.P.S. GROUPE MANE, France)	Beef burgers with addition of 0.5% pea fiber	Addition of pea fiber did not significantly affect the flavor and texture of the beef burger.	Consumer test (*n* = 36) using a 5‐point hedonic scale for flavor and texture (juiciness and appearance).	Besbes et al. 2008
Pea flour, pea fiber, and pea starch	Bologna sausages of different fat content with 4% pea flour, fiber, or starch (wheat flour as control)	Pea fiber and starch improved textural properties of low‐fat bologna sausage to become more similar to regular bologna. Pea flour exerted a negative impact on the texture and flavor.	Consumer tests (*n* = 74): affective acceptance using a 9‐point hedonic scale for appearance, flavor, moistness, firmness, and overall acceptability. Five‐point *just about right* (JAR) scales for moistness and firmness.	Pietrasik and Janz 2010
Pea fiber (Uptake 80), pea starch (Accu‐Gel), and pea flour (Century Yellow Pea Flour) from Nutri‐Pea Limited, Canada^c^	Breakfast sausage with 4% of pea fiber, pea starch, or pea flour	Fiber‐ and starch‐enriched sausages had similar acceptance (regarding overall acceptability, appearance, flavor, juiciness, and firmness) as the control sausage. Adding pea flour to the sausage compromised the overall acceptability and flavor.	Consumer tests (*n* = 79): affective acceptance using a 9‐point hedonic scale for appearance, flavor, juiciness, firmness, and overall acceptability.	(Pietrasik and Soladoye 2021a)
Pea fiber (ID 90, Campi y Jové, Spain)	Wheat flour bread supplemented with 2.5% fiber	Bread enriched with pea fiber did not significantly impede the palatability of wheat flour bread. Adding fiber enhanced the shelf life of the bread.	Consumer tests (*n* = 40): affective acceptance using a 9‐point hedonic scale for overall acceptability.	Gómez et al. 2003
Pea fiber (Juliá‐Parrera, Spain)	Wheat flour bread enriched with 3% pea fiber	Fiber‐enhanced bread was accepted by the consumer. Although liking of “grain,” “flavor,” and “overall acceptability” decreased, liking of the “crumb smoothness” and “aroma” increased.	Consumer tests (*n* = n.d.): affective acceptance using a 9‐point hedonic scale for quality attributes: grain, crumb smoothness, aroma, flavor, and overall acceptability.	J. Wang et al. 2002
Pea starch	Ingredient in formulation for coating of walnuts and pine nuts	Improved smoothness, taste, and overall acceptance in formulations with pea starch compared to those without.	Descriptive sensory test with a trained panel (*n* = 20) using a 9‐point hedonic scale for evaluating color, glossiness, smoothness, taste, smell, bite strength, crunchiness, and overall acceptance.	Mehyar et al. 2012
Modified commercial pea starch (Cosura S. A., Belgium)^d^	Bread enriched with modified pea starch (10%, 20%, 30%)	Inclusion of pea starch affected the perceived sensory properties of the bread. An inclusion level up to 20% was considered acceptable, although it deviated from the control and the bread supplemented with 10% pea starch. Overdosing (30% pea starch) had a negative impact on texture, odor, taste/flavor, and overall acceptability.	Trained analytical panel (*n* = n/a) using semistructured scales, from 1 to 10, to evaluate visual, textural, and organoleptic characteristics.	Sanz‐Penella et al. 2010
Pea starch (Purity P 1002, Ingredion, USA)	Pea starch–containing (10% and 20%) beverages	Particle size and starch concentration affect the chalky perception.	Consumer test (*n* = 82) evaluating intensity of chalky, powdery, gritty, sandy, mouth‐drying, and residual mouth‐coating.	K. K. Ma et al. 2024
Pea starch (Accu‐Gel, Nutri‐Pea Limited, Canada) and air‐classified pea starch (Starlite, Parrheim Foods, Canada)	Pea starch (3%)–enriched beef products	Adding air‐classified pea starch and wet‐extracted pea starch did not significantly impact the color, flavor, juiciness, firmness, texture, and overall acceptability. Only wet‐extracted pea starch deviated from the control regarding firmness.	Consumer panel (*n* = 81) test using 9‐point hedonic scales for evaluating color, flavor, juiciness, firmness, texture, and overall acceptability.	Pietrasik et al. 2012

^a^ Pea fiber from heat‐treated shells of yellow and green peas. Consisted of cellulose (48.5%), hemicellulose (18%), pectin (14%), lignin (2%), and other compounds (17.5%) according to the manufacturer data sheet. The total dietary fiber content was 90% (w/w).

^b^ Pea fiber fraction consisting of moisture (∼10%), fiber content (50% dry basis), protein content (maximum 10% dry basis), and starch (35% dry basis).

^c^ Uptake 80 is obtained from the kernel of non‐GMO Golden Canadian field peas. It consists of starch (50%), total dietary fiber (35%), protein (10%), fat (<0.5%), and ash (2% dry basis). Accu‐Gel is wet‐extracted from the kernel of yellow peas. It contains starch (95% dry basis), protein (1.0% dry basis), and ash (<0.2% dry basis). Yellow split pea flour contained carbohydrates (66% dry basis), protein (22% dry basis), and fat (<2% dry basis).

^d^ Commercial pea starch was used as a reference sample of raw material and for further modification. Commercial pea starch and modified pea starch consisted of moisture (11.6% and 16.2%, respectively), protein (0.19% and 0.13% dry basis), ash (0.11% and 0.48% dry basis), total starch (99.5% and 95.5% dry basis), and resistant starch (42.8% and 21.5% of total starch).

Several studies rely on untrained panels and uncontrolled environments, as shown in Table 5, which can impede the reliability and consistency of sensory analyses. For instance, 10 out of 15 studies used consumers as panelists. The most common approach is to perform an affective test, using the 9‐point hedonic scale to determine the liking of a specific product. It should be noted that there is a large variation in the number of consumers included in the described studies, with six being the lowest and 122 the highest. This implies large variations in statistical power as well as in trustworthiness (Schmidt et al. 2018). The other described studies used trained sensory panelists to profile the included products as objectively as possible. Typically, these panelists do not express personal opinions but assess the intensities (often in triplicate) of selected attributes on which they have received training. However, looking at Table 5, one of the studies with a trained panel utilized it for hedonic purposes, while another study used a consumer panel for descriptive purposes. The discrepancy in approaches complicates the comparison of different studies.

In addition to the sensory assessment settings, the sensory properties of pea starch and fibers are influenced by several other factors, such as physicochemical properties (affected by cultivation parameters, postharvest handling, extraction and modification processes, pea fraction, and purity), food matrices, and inclusion levels. Various statistical methods can be applied to investigate the relationships between these influencing factors and sensory properties, including regressions (linear, multiple, nonlinear) and correlations (Pearson, Kendall, Spearman). For instance, partial least squares regression (PLSR) has been used to study the quality of frozen green peas (Nleya et al. 2014) and the influence of storage conditions on green peas (Berger et al. 2007). Westling et al. (2024) linked sensory attributes to pea varieties by canonical variate analysis, which explained sensory variations in relation to the location or year of harvest. Similarly, García Arteaga et al. (2021) employed principal component analysis (PCA) and Kendall correlation analysis to assess the similarities among pea cultivars and their physicochemical, functional, and sensory properties. Further research and an enhanced understanding of sensory methodology are necessary to gain a deeper understanding of the relationship between various physicochemical properties and sensory characteristics of peas and pea fractions.

## 5 Current Challenges and Future Opportunities

The literature cited in this review shows that pea starch is a versatile ingredient that can be used in a range of products due to its functionalities. These include its digestive properties, high RS content, and low GI value (Krause, Debon, et al. 2022; Z.‐H. Lu et al. 2022; Ren, Yuan, et al. 2021; Sanz‐Penella et al. 2010); stabilizing effect on product structure, firm gel formation, and strong binding ability (Morales‐Hernández et al. 2024; Ren, Yuan, et al. 2021; Pietrasik et al. 2020; Pietrasik and Soladoye 2021b; N. Wang et al. 2012; Sanz‐Penella et al. 2010); and a neutral aroma and a low number of volatiles (Krause, Asamoah, et al. 2022). When pea starch is incorporated into foods, it is important to be aware of the physicochemical changes it undergoes during processing to understand how it will impact the microstructure and product texture. If pea starch is used to replace other starchy ingredients, knowing the differences between the physicochemical properties and their effect on texture is critical to finding the ideal replacement ratio and how to tailor processing methods for optimal texture. The texturizing effect of pea starch may also be further optimized by modifying the starch macromolecular structure or by using different processing techniques to promote specific physicochemical changes.

Both the texturizing effect and the digestive properties of pea starch relate to its macromolecular structure and granular morphology. For the development of functional ingredients, it is important to understand the correlations and discrepancies between the two variables. For instance, Ciardullo et al. (2019) found that the macromolecular properties and processing variables during extrusion (that enhanced the RS content of pea starch) reduced the viscosities of the pea starch mixtures and their textural suitability as an extrudate. For the development of functional ingredients, it could be interesting to find modification/processing methods that enhance the RS content of the pea starch, without negatively affecting its strong texturizing effects.

Based on current knowledge and practical experience, significant challenges remain in modifying the properties of pea fibers, particularly the more rigid fractions derived from pea hulls and pods. For instance, pea cotyledon fibers are generally more susceptible to enzymatic treatment, whereas pea hull fibers often require additional pretreatments to achieve comparable levels of enzymatic breakdown. Another difficulty when it comes to enzymatic breakdown is the control of the molar mass of the solubilized fibers and its effect on the technofunctional properties of the fibers. Technofunctional properties require careful control of the molar mass of the degradation products, tuned for specific applications. For example, extensive degradation of the polysaccharide into mono‐ and small oligosaccharides may not increase the technical functionality of the fibers in wet products but may be beneficial in dry. Additionally, while the rigidity and limited functionality of pea hull fibers can restrict their use in certain applications, the pea fibers have demonstrated potential for incorporation into bread formulations without compromising sensory quality.

Many challenges of incorporating pea fibers in different products relate to the large particle size and insolubility. This could lead to issues with sedimentation in beverages and dispersions, as well as grittiness due to the large particles. Hence, treatments of pea fibers have often focused on reducing the particle size. Further research should continue to explore the effects of particle size on the physicochemical properties of pea fibers and try to elucidate the mechanism behind these changes.

While it is well established that many of the treatment methods discussed in this review affect the structure, particle size, and morphology of pea fibers, their effect on the molecular structure remains less understood. Increased knowledge at the molecular level would be beneficial but is challenging due to the inherent complexity of pea fibers and plant cell walls in general. This complexity arises from several factors, including the diversity and molecular weight distribution of polysaccharides, the insolubility of cellulosic fractions, variations in the degree of crystallinity, variations in the abundance and structure of side chains, the presence of additional components such as lignin and proteins, and the extensive interactions among these constituents. Furthermore, elucidating complete molecular structures is technically difficult, as analyses typically rely on hydrolysis prior to characterization.

Due to the limited number of studies on the topic, future studies could also continue exploring the use of enzymes and combined treatments of pea fibers. The study by Morales‐Medina et al. (2024) showed the potential of using enzymatic treatments before microfluidization. Further research could explore combining enzymatic pretreatments with other physical methods, such as steam explosion. Treatments that have been used to modify dietary fiber but not yet for peas include microwave treatment, steam explosion, and fermentation by microorganisms (Gan et al. 2021). Both microwave treatment and steam explosion are emerging techniques used to modify dietary fiber and have shown the potential to modify the properties of fibers from other sources (Gan et al. 2021).

Additionally, it is important to acknowledge and further investigate the differing functionality of fibers obtained from the cotyledon, hull, and pod of the pea to find the source most suitable for specific needs and applications. The source will also influence the effect of different processing treatments and should be clearly stated (and preferably characterized) in future studies. For now, most studies investigating the effect of physical, chemical, or enzymatic treatments on pea fibers have focused on fibers obtained from the pea hull. Due to its high fiber content and large volumes handled during pea processing (Bento et al. 2025), pea pods can become a useful food ingredient if suitable processing treatments and food applications can be found. A few studies have investigated the effect of pea pod powder on different foods, such as bread and mayonnaise (Nasir et al. 2024). However, further research is needed to properly evaluate pea pods for different food applications and how chemical, physical, and enzymatic treatments may enhance their functionality.

Furthermore, one of the main purposes of modifying pea starch and pea fiber is to alter the functionality for improved use in different food products and open the way to more efficient utilization of side streams. Hence, it is important to investigate how changes in functionality affect the textural and structural properties of the final food product and its sensory properties. Accordingly, future research should also continue to explore how changes in starch and fiber properties affect the properties of the final food product and not just the physicochemical properties of the starch and fiber themselves.

When navigating the available literature on sensory studies on peas and pea fractions, several constraints in the methodologies are observed, which may affect the overall knowledge. For instance, descriptive sensory analysis is not always pursued in controlled environments using a trained panel. Opting for trained panelists over untrained ones provides a more consistent and reliable approach to sensory analyses. This was also discussed by Lara and Tsiami (2024). Untrained panels could be used in affective sensory studies for which consumer input is valuable. There is also a general misconception regarding different sensory terms. For example, gustatory (taste) and olfactory (smell) senses jointly create the perception of flavor but are sometimes mixed up. This creates confusion as to what is actually being perceived. In general, there is a lack of chemical correlations to the sensory properties, which may be valuable when trying to understand new products in depth. Finally, regarding pea starch and fiber in particular, in the papers that were analyzed, these fractions were rarely pure and could hence be complemented with data on their composition and extraction method.

Finally, multiple challenges exist related to the commercialization of pea starch and pea fiber. These may include higher production costs compared to nontreated starch/fiber from other sources, consumer acceptance of upcycled or chemically modified ingredients, and regulations that might apply to some of the more novel treatment methods. These issues need to be addressed before starch and fiber from peas can reach their full market potential.

## 6 Conclusions

This review has summarized current literature on pea starch and pea fiber, plus side streams from pea protein extraction, focusing on their functional and sensory properties in food applications and how they are affected by different chemical, physical, and enzymatic modification methods. Both pea starch and pea fiber have been shown to have multiple applications across a range of different foods. These ingredients provide an opportunity to reduce waste while acting as functional ingredients for improved physicochemical properties or nutritional value. Their physicochemical properties can be modified and enhanced using various physical, chemical, and/or enzymatic treatments, further expanding the uses of these pea‐derived components.

The high amylose content of pea starch results in properties and potential uses that may complement the more commonly used potato and cereal starches. The high amount of RS typically found in pea starches may also provide additional health benefits. For pea fiber, the current literature suggests that physical treatments appear to be the most effective methods for modifying pea fibers, as they induce the most substantial changes in functionality. Furthermore, physical treatments are often preferred due to low cost and reduced need for chemicals. Combined treatments may further alter fiber properties, but additional research is required to fully understand their effects and potential synergies.

Evaluation of texture and sensory properties of diverse food products with added pea starch or pea fiber has demonstrated both potential benefits and limitations, depending on the inclusion level. Some of the main obstacles include formulation challenges for the starch and the insolubility of the fiber. These challenges may be mitigated through targeted modification techniques, tailored to the specific requirements of different food formulations, and the versatile use of pea fibers and pea starch overall highlights their usefulness within the food industry. Further research is still needed to better understand the relationship between the physicochemical properties of peas and their sensory characteristics. In particular, studies examining how various modification techniques influence sensory properties remain limited and warrant greater attention.

## Author Contributions

**Mathias Johansson**: conceptualization, investigation, visualization, writing – original draft. **Klara Nilsson**: writing – original draft, conceptualization, investigation. **Madeleine Jönsson**: writing – original draft, conceptualization, investigation. **Karin Wendin**: funding acquisition, supervision, writing – review and editing. **Anna Ström**: writing – review and editing, supervision, funding acquisition. **Maud Langton**: funding acquisition, writing – review and editing, project administration, supervision.

## Funding

This work was supported by the Swedish Research Council Formas (grant number 2023‐01906).

## Conflicts of Interest

The authors declare no conflicts of interest.
